# Study on the Rheological Properties of Recycled Plastic and Waste Cooking Oil Composite Modified Asphalt

**DOI:** 10.3390/ma18204762

**Published:** 2025-10-17

**Authors:** Maowen Li, Ping Zheng, Chao Pu, Dongxu Xu, Waiti Litifu, Zhe Ma, Peng Yin

**Affiliations:** 1 Xinjiang Transportation Investment (Group) Co., LTD., Urumqi 830011, China; 2 Xinjiang Key Laboratory for Safety and Health of Transportation Infrastructure in Alpine and High-Altitude Mountainous Areas, Urumqi 830011, China; 3 Xinjiang Transport Planning Survey and Design Institute Co., Ltd., Urumqi 830011, China; 4 School of Infrastructure Engineering, Dalian University of Technology, No. 2, Linggong Road, Ganjingzi District, Dalian 116024, China; 5 Xinjiang Road and Bridge Construction Group Co., LTD., Urumqi 830011, China; 6 Xinjiang Communications Investment Construction Management Co., Ltd., Urumqi 830011, China

**Keywords:** modified asphalt, recycled plastic, waste cooking oil, rheological properties, modification mechanism

## Abstract

To enhance the overall performance of asphalt pavements and promote the efficient utilization of solid waste resources, this study innovatively incorporates recycled polyethylene (PE) particles and recycled ethylene-vinyl acetate copolymer (EVA) particles, each compounded with waste cooking oil (WCO), to modify base asphalt. Systematic tests were conducted to evaluate the physical and rheological properties of the composite modified asphalt. Additionally, Fourier transform infrared spectroscopy (FTIR) and thin-layer chromatography with flame ionization detection (TLC-FID) were used to analyze the microstructures and internal components of the modified asphalt. The results indicate that the optimal mixing ratio for the WPA is 5% WCO, 5% EVA, and 5% PE. With the incorporation of these modified materials, the asphalt’s high-temperature and low-temperature properties, as well as its rutting and fatigue resistance, are enhanced to some extent. Furthermore, the modification significantly improves the rheological properties of the asphalt across the full temperature range. Additionally, the modified materials lead to changes in the internal composition of the asphalt: the content of lighter components decreases, while the content of heavier components increases. These changes in the internal composition are the primary cause of the observed improvements in the rheological properties of the asphalt.

## 1. Introduction

With the proposal of the “dual carbon” goals, green and low-carbon development in the transportation sector has become a key trend, and the efficient recycling of waste resources is a crucial measure to achieve this goal [1]. A large amount of waste, such as waste plastics and waste cooking oil (gutter oil), is generated in daily life [2,3]. If these wastes cannot be properly disposed of, they will not only cause a waste of resources but also pollute the environment. Applying these waste resources to asphalt modification is a highly valuable exploration path [4]. This approach can not only realize the “turning waste into treasure” of waste, reduce reliance on resources such as virgin petroleum asphalt, and conform to the concept of green development, but also effectively improve the high-temperature and low-temperature performance and anti-aging performance of asphalt through scientific modification processes, making asphalt pavements more durable [5]. Therefore, actively exploring the synergistic modification technology of waste resources and asphalt in highway engineering is not only an inevitable requirement for promoting the green transformation of the transportation industry, but also an effective way to improve pavement quality and reduce the whole-life-cycle cost, which has important practical significance [6].

Recycled plastic particles are derived from the recycling and processing of waste plastics [7]. In recent years, some scholars have attempted to incorporate them into asphalt materials, aiming to improve the service performance of asphalt through this approach [8]. Boom [9] introduced recycled plastics and polyethylene into traditional hot-mix asphalt, and evaluated the mechanical properties of the modified asphalt. The results showed that the fatigue performance and rutting resistance of the modified asphalt were significantly enhanced; moreover, the use of the modified asphalt significantly reduced the gas emissions from the two types of recycled plastics, but the low-temperature performance of the asphalt decreased to some extent. Lu [10] explored the feasibility of recycling plastic-modified asphalt at the end of its service life. Recycled plastic-modified asphalt was reused in a certain proportion to prepare recycled asphalt mixtures, and the crack resistance, fatigue performance, and rutting performance of the mixtures were analyzed. The results showed that the recycled plastic-modified asphalt mixtures were fully recyclable, and the performance of the recycled mixtures basically met the service requirements. Dalhat [11] studied the effect of recycled plastic waste on the viscoelastic properties of asphalt, and found that an increase in recycled plastic waste effectively improved the performance grade of asphalt, and the rutting performance and fatigue performance of the modified asphalt were also significantly enhanced. Hao [12] investigated the influence of different types of plastics on the long-term performance of asphalt mixtures, and found that except for low-temperature crack resistance, recycled plastic-modified asphalt mixtures generally exhibited better long-term performance; however, when the plastic content exceeded 15%, the long-term performance of the mixtures decreased. Melo [13] used fragments of latex gloves as a modifier to prepare modified asphalt and found that they effectively enhanced the high-temperature performance of the asphalt. When the content reached 12%, the rutting deformation of the asphalt was reduced by 38%. The use of latex glove fragments significantly improved the service performance of the asphalt at high temperatures. Ma [14] explored the effect of recycled plastics on asphalt performance, and found that the incorporation of recycled plastics effectively improved the physical properties of asphalt, but the low-temperature performance was affected to a certain extent. In addition, the incorporation of rubber could further improve the compatibility of recycled plastic-modified asphalt, which was conducive to promoting the commercial application of modified asphalt. It can be concluded that although plastic-modified asphalt improves the high-temperature performance and fatigue performance of asphalt, it has an adverse effect on the low-temperature performance of asphalt. Therefore, the application of recycled plastics in asphalt still requires further research.

WCO has a wide range of sources, with an annual output of approximately 4 million tons in China. If a large amount of such waste is not properly treated, it will not only cause waste accumulation problems but also severely pollute the environment. Therefore, recycling it as an asphalt modifier is an effective waste disposal method, with significant economic and social benefits [15]. Li [16] applied waste cooking oil to recycled aged asphalt and found that it could effectively soften the aged asphalt and restore its workability, especially significantly improving the low-temperature performance of the aged asphalt. Zhong [17] explored the influence of waste cooking oil on the physical and rheological properties of aged asphalt and found that it could restore the performance of most aged asphalt, particularly in terms of low-temperature ductility; however, when its content reached a certain threshold, the restoration effect on low-temperature performance was limited. Bardella [18] innovatively used waste cooking oil as a regenerant for recycled asphalt mixtures and found that its incorporation could effectively improve the low-temperature crack resistance of the mixtures, and the overall performance of the recycled mixtures could meet service requirements, further verifying the application value of waste cooking oil. Mamun [19] evaluated the effect of different contents of waste oil on the performance of recycled asphalt mixtures and found that the performance of the mixtures was significantly restored, and the use of waste oil effectively improved low-temperature performance. From the above research results, waste cooking oil can effectively improve the low-temperature performance of asphalt. However, the previous research results indicate that although recycled plastics can improve the high-temperature and fatigue performance of asphalt, they have an adverse effect on low-temperature performance. Therefore, if recycled plastics and waste cooking oil can be used together for asphalt modification, it is expected to comprehensively improve the overall service performance of asphalt while further promoting the resource utilization of these two types of solid waste.

To address the poor low-temperature performance of asphalt caused by plastic modification alone, this study innovatively uses waste cooking oil for synergistic asphalt modification. This study aims to evaluate the rheological, physical, and structural performance of asphalts modified with recycled plastics and waste cooking oil, aiming to propose a sustainable formulation with a better balance between performance at high and low temperatures. For this purpose, this study adopts waste recycled plastics and waste cooking oil to conduct composite modification on asphalt. Subsequently, physical tests, rheological tests, and microscopic tests are carried out to evaluate and analyze the overall service performance of the modified asphalt, hoping to provide some references for the resource utilization of solid waste in asphalt pavements.

## 2. Materials and Methods

### 2.1. Materials

The virgin asphalt (VA) used in this study is of the penetration grade 80–100, and its properties are presented in Table 1. The WCO employed is derived from waste oil after frying, which has undergone impurity screening and treatment; its properties are listed in Table 2. The recycled plastics used include polyethylene (PE) and ethylene-vinyl acetate copolymer (EVA) from Shanghai Xinshuofa Plastic Co., Ltd. in Shanghai, China. Specifically, PE particles feature low density and low strength but exhibit good elongation and cold resistance; EVA, as a general-purpose polymer, possesses excellent low-temperature resistance. The properties of these plastics are shown in Table 2.

### 2.2. Methods

#### 2.2.1. Orthogonal Test

To improve the service performance of asphalt and realize the recycling of plastics and waste cooking oil, this study used WCO, PE, and EVA to prepare composite modified asphalt. Studies have shown that WCO contains long-chain fatty acid glycerides, which can improve the low-temperature brittleness of asphalt and alleviate low-temperature cracking of pavements; PE, relying on its polymer chain structure, can enhance the high-temperature deformation resistance of asphalt and reduce the risk of rutting in heavy-load road sections; EVA, by virtue of the polarity of vinyl acetate groups, balances the flexibility of WCO and the rigidity of PE, while improving the interfacial adhesion between asphalt and aggregates. The complementary properties of the three materials provide feasibility for the development of composite modified asphalt that balances solid waste resource utilization and service performance. Based on this, an orthogonal experiment was adopted in this study to optimize the formula. According to relevant research experience [30,31], the dosage range of WCO was set to 5–9%, that of EVA to 3–5%, and that of PE to 4–6%. The specific factor levels are shown in Table 3.

#### 2.2.2. Preparation Process of Modified Asphalt

Based on relevant research experience and preliminary exploratory test results [32,33], the preparation process of modified asphalt was determined as follows: The VA was heated to 160 °C, after which a certain amount of EVA and PE were added. A high-speed shearing machine was used to shear the mixture at 1500 r/min for 30 min. Subsequently, a specified amount of WCO was added to the sheared mixture, which was then sheared again at 1500 r/min for 20 min using the high-speed shearing machine(JRH200) from Shanghai Leiqi Instrument & Equipment Co., LTD., Shanghai, China. Finally, the mixture was incubated at 160 °C for 10 min to obtain the composite modified asphalt of recycled plastics and WCO, which was named WPA.

#### 2.2.3. Physical Property Tests

To characterize the effects of several modified materials on the physical properties of WPA, this study conducted tests on the penetration, ductility, softening point, and viscosity of the modified asphalt in accordance with the specifications of JTG E20-2011 [20], the test methods were carried out in compliance with relevant procedures, and each sample was subjected to three repeated tests.

#### 2.2.4. Rheological Tests

Temperature sweep test: To reflect the effects of several modified materials on the high-temperature performance of WPA, a dynamic shear rheometer (DSR) was used to characterize the high-temperature deformation resistance of the modified asphalt. In accordance with JTG E20-2011, the test was conducted under the strain-controlled mode with a controlled strain of 12%, and the test temperatures ranged from 46 °C to 76 °C at an interval of 6 °C, and each sample was subjected to three repeated tests.

Multiple stress creep recovery (MSCR) test: To further evaluate the rutting resistance of the modified asphalt in high-temperature environments, the MSCR test was adopted to assess the rutting resistance of WPA. In line with AASHTO T350-14 [34], the test was performed at a temperature of 64 °C with stress levels of 0.1 kPa and 3.2 kPa, and each sample was subjected to three repeated tests.

Linear amplitude sweep (LAS) test: To evaluate the fatigue performance of WPA, the LAS test was used to analyze the fatigue performance of asphalt before and after modification. According to AASHTO TP 101-14 [35], a frequency sweep was first conducted at a strain of 0.1% with frequencies ranging from 0.2 Hz to 30 Hz, followed by the LAS test at a frequency of 10 Hz within a linear amplitude range of 0.1% to 30%. The test temperature was set at 25 °C, and each sample was subjected to three repeated tests.

Bending beam rheometer (BBR) test: The low-temperature performance of the modified asphalt was evaluated via the BBR test in this study. In accordance with AASHTO T313 [36], the test was carried out at temperatures of −6 °C, −12 °C, and −18 °C, respectively, and each sample was subjected to three repeated tests.

#### 2.2.5. FTIR Test

To evaluate the interaction mechanism between several materials and VA, FTIR tests were conducted on asphalt before and after modification. In accordance with GB/T 6040-2019 [37], the test range was set to 4000–400 cm^−1^, and the resolution was 4 cm^−1^, and each sample was subjected to three repeated tests.

#### 2.2.6. TLC/FID Test

The TLC/FID test was used to test the four components of asphalt before and after modification. In accordance with JTG 3410-2025 [38], the TLC/FID test selects a developing solvent with matching polarity based on the polarity differences in each component to separate the components. After that, the components are scanned via flame ionization detection; this process generates peak graphs and peak areas of each chemical component, which are then used to calculate the chemical content of each component, and each sample was subjected to three repeated tests.

## 3. Results

### 3.1. Development of Modified Asphalt

To explore the optimal composition ratio of the modified materials in WPA, this study used an orthogonal experiment to investigate the formula of WPA [39,40]. Penetration, ductility, softening point, and viscosity were selected as the evaluation indicators, and the results are presented in Table 4.

Table 4 presents the test results of the orthogonal experiment and the calculation results of the intuitive analysis. It can be observed that compared with VA, as the dosages of EVA and PE increase, the penetration of the composite modified asphalts gradually decreases, while their ductility and softening point gradually increase. In contrast, with the increase in WCO dosage, the penetration of the composite modified asphalts increases gradually, whereas their ductility and softening point decrease progressively. This is because EVA and PE are essentially elastoplastic polymer materials. After shear mixing with asphalt, they tend to form a relatively stable network structure inside the asphalt matrix. This network structure enhances the asphalt’s resistance to deformation at high temperatures and improves its ductility at low temperatures. However, as the dosages of EVA and PE increase, the viscosity of the asphalt also increases gradually, and its stiffness is enhanced accordingly—ultimately leading to a gradual reduction in penetration. As WCO is gradually incorporated into the asphalt matrix, the content of light components inside the asphalt increases, leading to gradual softening of the asphalt. Consequently, the penetration of the asphalt decreases. However, with the progressive enhancement of asphalt softening, the asphalt’s resistance to deformation at high temperatures is gradually lost, and its ductility at low temperatures is also adversely affected. Macroscopically, these changes manifest as a decline in the softening point and ductility of the modified asphalt. It is worth noting that the softening point and ductility can only characterize the high and low temperature performance of asphalt to a certain extent; their changes cannot fully reflect the variation trend of the asphalt’s high and low temperature performance. Further investigations into the high and low temperature performance will be conducted in the subsequent temperature sweep test and BBR test sections of this study.

Interestingly, in the intuitive analysis of several performance indicators, A_1_ and B_3_ were generally identified as the optimal dosages for WCO and EVA, respectively; however, the recommended dosage for PE was not clearly indicated. To address this ambiguity, this study conducted experimental analyses on the composite modified asphalt under three different mix ratios: A_1_B_3_C_1_, A_1_B_3_C_2_, and A_1_B_3_C_3_. The results are presented in Table 5. As shown in Table 5, an increase in PE dosage leads to a gradual increase in ductility and softening point, while the penetration decreases progressively. Moreover, an excessively high PE dosage results in excessively high viscosity of the modified asphalt, which in turn impairs its viscoelastic properties. Through comprehensive analysis, it was found that when the mix ratio of the composite modifier is A_1_B_3_C_2_, the composite modified asphalt achieves relatively optimal test results. Therefore, based on the results of the orthogonal experiment and relevant exploratory tests, this study recommends the formula of the composite modified agent as follows: 5% WCO, 5% EVA, and 5% PE. Additionally, the independently developed composite modified asphalt was named WPA.

### 3.2. Rheological Tests

#### 3.2.1. Temperature Sweep Test

**Figure 1:**
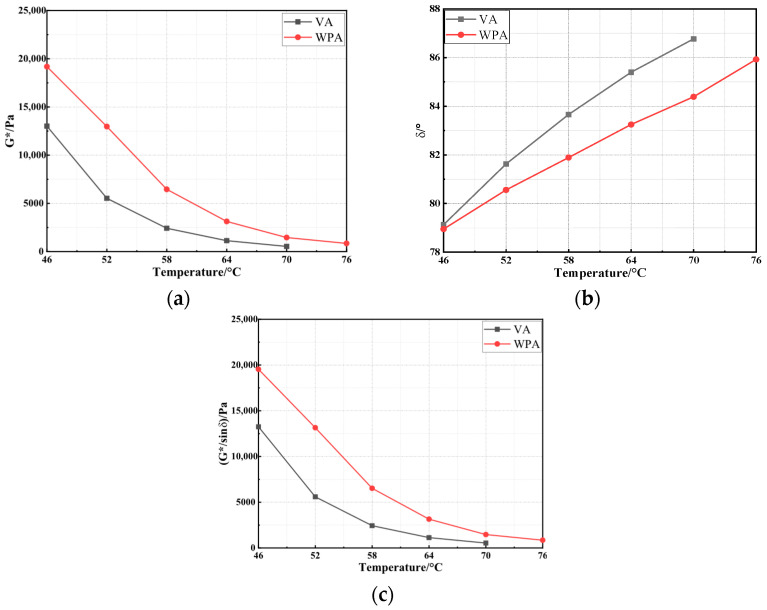
The results of temperature sweep test: (**a**) G*; (**b**) δ; (**c**) G*/sinδ.

In this study, the temperature sweep test was conducted to investigate the variation trend of the high-temperature performance of asphalt before and after modification. The complex modulus (G*), phase angle (δ), and rutting factor (G*/sinδ) of the asphalts were calculated to further evaluate the high-temperature performance of WPA [41]. The results are presented in Figure 1.

As shown in Figure 1, with the gradual increase in temperature, the G* increases progressively while the δ decreases gradually. Among them, the G* represents the overall stiffness of the material, reflecting its resistance to deformation, while δ indicates the ratio of the material’s elastic to viscous components, revealing its rheological characteristics. Notably, a higher G* value and a lower δ value indicate better high-temperature deformation resistance of asphalt. This phenomenon demonstrates that increasing temperature leads to a gradual loss of the asphalt’s high-temperature deformation resistance—i.e., elevated temperatures result in the degradation of asphalt’s high-temperature performance. Furthermore, it can be observed that compared with VA, WPA exhibits a higher G* value and a lower δ value. This indicates that the high-temperature performance of asphalt is significantly improved after composite modification. The underlying reason is that EVA and PE are essentially polymeric materials; when uniformly mixed with asphalt, they tend to form a relatively stable network structure within the asphalt matrix. This structure provides more significant resistance under high-temperature and shear conditions, thereby endowing WPA with more reliable high-temperature performance, which is in line with the findings of Yu [42]. To further evaluate the performance grade (PG) of asphalt before and after modification, this study calculated the G*/sinδ of the two types of asphalt, with the results presented in Figure 1c. As shown in Figure 1c, when the test temperature reaches 70 °C, the G*/sinδ value of VA drops below 1 kPa; in contrast, the G*/sinδ value of WPA does not fall below 1 kPa until the temperature reaches 76 °C. This indicates that the high-temperature PG of VA is PG 64, while that of WPA is PG 70. This result is consistent with the analysis conclusions from G* and δ, further verifying that the composite modification effectively improves the high-temperature performance of VA.

#### 3.2.2. MSCR TEST

**Figure 2:**
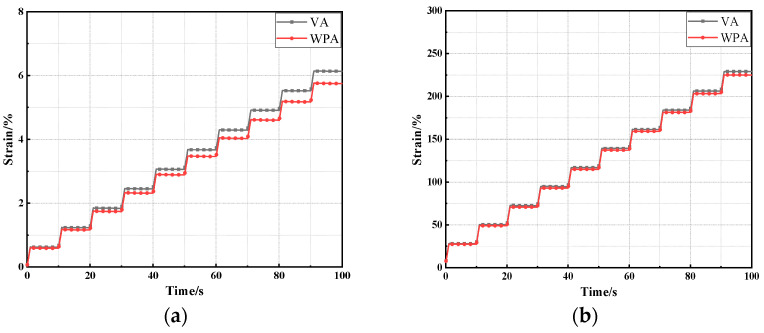
The MSCR test results of several asphalts: (**a**) 0.1 kPa; (**b**) 3.2 kPa.

To further characterize the rutting resistance of asphalt before and after modification, thereby further evaluate the effect of composite modification, this study conducted MSCR tests on both unmodified and modified asphalt [43]. The results are presented in Figure 2.

As shown in Figure 2, under all stress levels, the MSCR curves exhibit a consistent pattern: with the increase in loading time, the cumulative strain of both VA and WPA gradually increases. Moreover, when the stress level rises, the cumulative strain increases further and significantly. In the performance system of asphalt materials, the magnitude of cumulative strain is closely related to rutting resistance. A larger cumulative strain indicates more obvious permanent deformation of asphalt during load bearing, which means its rutting resistance is poorer. It can thus be concluded that an increase in stress level exerts an adverse effect on the rutting resistance of asphalt. This aligns with the practical phenomenon in road engineering where rutting distress is more prominent in road sections subjected to heavy traffic loads. A further comparison between the curves of VA and WPA reveals that under the same loading time and stress level, the cumulative strain of WPA is significantly lower than that of VA. This is attributed to the large amount of EVA and PE added to WPA, EVA possesses excellent flexibility and adhesion, while PE exhibits high strength and thermal stability. These two polymers interact with each other inside the asphalt matrix and tend to form a relatively stable network structure. This network structure can effectively restrict the flow and deformation of asphalt components at high temperatures, thereby significantly enhancing the asphalt’s deformation resistance in high-temperature environments—and ultimately resulting in a smaller cumulative strain of WPA under load. This conclusion is also consistent with the results of the temperature sweep test conducted earlier.

**Figure 3:**
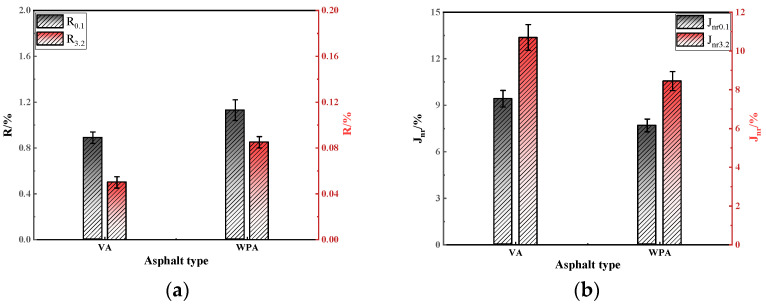
The calculation results of MSCR index of several asphalts: (**a**) R; (**b**) Jnr.

In addition, to further quantitatively analyze the variation trend of asphalt’s rutting resistance before and after modification, this study calculated the creep recovery rate (R) and non-recoverable creep compliance (Jnr) of several asphalts under different stress levels. The results are presented in Figure 3.

As shown in Figure 3, under the two stress levels, the R and Jnr of both VA and WPA exhibit a similar variation pattern, as the stress level increases from 0.1 kPa to 3.2 kPa, the R values of both asphalts gradually decrease, while their Jnr values gradually increase. Among them, R represents the percent recovery, indicating the asphalt’s ability to recover after deformation, while Jnr measures the asphalt’s permanent deformation under repeated loading, reflecting its rutting resistance. R and Jnr are key indicators for characterizing asphalt’s rutting resistance. Specifically, a higher R value and a lower Jnr value indicate better rutting resistance of asphalt. This phenomenon demonstrates that an increase in stress level leads to the degradation of asphalt’s rutting resistance. This is because excessively high stress accelerates the deformation process inside asphalt, making the asphalt more prone to irreversible plastic deformation, and consequently leading to a decline in rutting resistance. Furthermore, a comparison of the performance between VA and WPA shows that WPA has an overall higher R value and a lower Jnr value than VA, indicating that WPA’s rutting resistance is superior to that of VA. The reason lies in the large amount of EVA and PE contained in WPA, when blended with asphalt, the two polymers tend to form a relatively stable three-dimensional network structure inside the asphalt matrix. This network structure enhances the structural stability of the asphalt and improves its resistance to load-induced effects, thereby effectively reducing the occurrence of irreversible deformation, which is in line with the findings of Alghrafy [44]. As a result, WPA exhibits better rutting resistance, which is also consistent with the results of related studies such as the temperature sweep test. In summary, WPA has a significant advantage over VA in terms of rutting resistance, which is more conducive to improving the durability of asphalt pavements under high-temperature and load conditions.

#### 3.2.3. LAS Test

**Figure 4:**
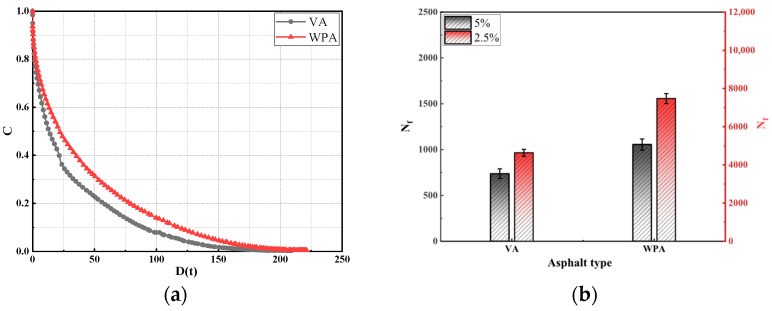
The LAS test results of several asphalts: (**a**) C; (**b**) N_f_.

During the long-term service life of asphalt pavements, sufficient fatigue performance is required to resist the cyclic and repeated action of vehicle loads. Theoretically, WPA should exhibit more prominent fatigue performance due to the EVA and PE it contains. To systematically evaluate the effect of modification on asphalt fatigue performance, this study took asphalts as the research object and conducted LAS test [45]. The fatigue performance was assessed using three indicators: the integrity parameter (C), the damage factor (D(t)), and the fatigue life (Nf). The results are presented in Figure 4.

As shown in Figure 4, for both VA and WPA, the decay rate of the C exhibits different patterns as the D(t) increases, specifically, the decay rate of C for WPA is slower than that for VA. Among them, C represents the creep compliance, D(t) is the time-dependent deformation under stress, and N_f_ is the number of loading cycles to failure, all used to evaluate the asphalt’s resistance to permanent deformation and fatigue cracking. It can thus be seen that compared with VA, WPA is more effective in enhancing the fatigue performance of asphalt. This is because WPA contains a large amount of EVA and PE; during the blending process with asphalt, these two polymers interact with each other and form a stable three-dimensional interwoven network within the asphalt matrix. This network structure further improves the asphalt’s resistance to shear failure, thereby contributing to its superior fatigue performance. As shown in Figure 4b, under strain levels of 2.5% and 5%, the N_f_ of WPA is higher than that of VA. A larger N_f_ value indicates a stronger ability of asphalt to resist fatigue failure, which demonstrates that the incorporation of WPA is beneficial for improving the fatigue performance of asphalt, which is consistent with the patterns reflected by the integrity parameter (C) and damage factor D(t). Meanwhile, when comparing N_f_ values under different strain levels, it is observed that as the strain increases from 2.5% to 5%, the N_f_ of both VA and WPA gradually decreases. This is because excessively high strain causes asphalt to enter the fatigue failure stage rapidly, thereby leading to a decline in N_f_. In summary, the incorporation of WPA can effectively enhance the fatigue performance of asphalt. The stable network structure formed by EVA and PE in WPA improves the asphalt’s resistance to shear damage and fatigue failure, and this enhancement effect is manifested under different strain levels, which is in line with the findings of Zeiada [46]. However, the magnitude of strain affects fatigue life: high strain accelerates the process of fatigue failure.

#### 3.2.4. BBR Test

**Figure 5:**
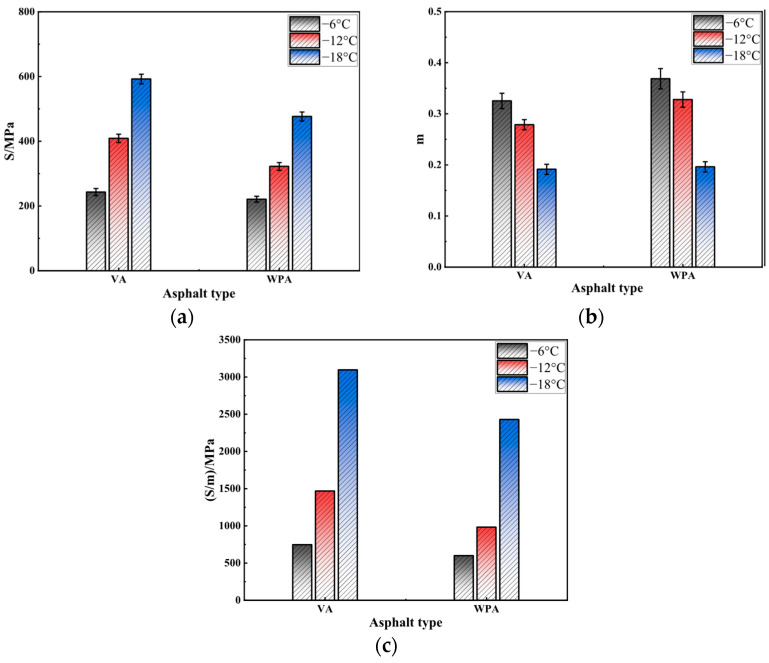
The results of BBR test: (**a**) S; (**b**) m; (**c**) S/m.

Low-temperature performance is one of the key technical indicators that determine the serviceability and service life of asphalt pavements. This is particularly true in seasonal freeze–thaw regions or harsh cold climates, where low-temperature performance has a more prominent impact on the structural stability of pavements. To evaluate the effect of modification on the low-temperature performance of asphalt, this study conducted BBR tests on several asphalts [47]. The low-temperature performance was assessed using two key parameters: creep stiffness (S) and creep rate (m). The results are presented in Figure 5.

As shown in Figure 5, the low-temperature performance of asphalt is significantly affected by both temperature and asphalt type. As the temperature decreases from −6 °C to −18 °C, the S of both VA and WPA gradually increases, while their m gradually decreases. According to the AASHTO M320 specification (requiring m ≥ 0.3 and S ≤ 300 MPa), a smaller S value and a larger m value indicate better low-temperature performance of asphalt. Among them, S represents the stiffness of the asphalt binder at a given temperature, indicating its resistance to deformation, while m reflects the asphalt’s ability to relax stress and its susceptibility to low-temperature cracking. It can be observed that at −6 °C and −12 °C, most of the performance indicators of VA and WPA meet the specification requirements. However, when the temperature drops to −18 °C, the S of both VA and WPA increases significantly and their m decreases noticeably, with the indicator values no longer meeting the specification requirements. This indicates that the minimum temperature at which VA and WPA can meet the service requirements is higher than −12 °C. When comparing VA and WPA at the same temperature, WPA exhibits a lower S and a higher m than VA, indicating that WPA has superior low-temperature performance to VA. This is because WPA contains a large amount of EVA and PE; these two polymers tend to form a relatively stable network structure within the asphalt matrix, which enhances the asphalt’s deformability at low temperatures and reduces the occurrence of brittle fracture, thereby improving its low-temperature performance, which is in line with the findings of Bastola [48]. Furthermore, considering the S/m results in Figure 5c, their variation pattern is similar to that of S, under different temperatures, the S/m value of WPA is consistently lower than that of VA. This further verifies the conclusion that WPA has better low-temperature performance. In summary, a decrease in temperature degrades the low-temperature performance of asphalt. However, compared with VA, WPA—relying on the stable network structure formed by EVA and PE inside, can effectively improve properties such as low-temperature crack resistance, making it more suitable for the service requirements of asphalt pavements in low-temperature environments.

### 3.3. FTIR Test

**Figure 6:**
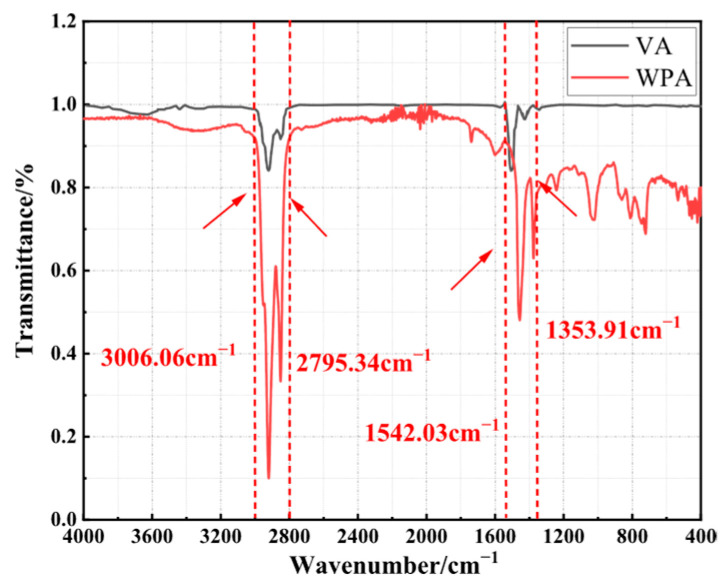
The FTIR test result of several asphalts.

The changes in the chemical structure of asphalt will inevitably lead to changes in its service performance. To further evaluate the effect of composite modification on the chemical structure of asphalt, this study conducted FTIR test on asphalt before and after modification. The results are presented in Figure 6.

As shown in Figure 6, both VA and WPA exhibit significant characteristic absorption peaks within the wavenumber ranges of 2795.34~3006.06 cm^−1^ and 1353.91~1542.03 cm^−1^. According to the analytical principle of infrared spectroscopy, these characteristic peaks are attributed to the stretching vibrations and out-of-plane bending vibrations of C-H bonds in asphalt molecules. Such vibrational behaviors have a strong correlation with the asphaltene content in asphalt; therefore, the difference in asphaltene content can be characterized by the intensity changes in these characteristic peaks. Focusing on the 0~1353.91 cm^−1^ waveband, the infrared spectrum of WPA shows distinct characteristic behaviors compared with that of VA. Within this waveband, both the relative intensity and peak shape of the absorption peaks of WPA have changed, which is related to the molecular structure vibrations of EVA and PE contained in WPA. The ester group (-COO-) in EVA molecules and the long-chain alkyl structure in PE molecules produce corresponding characteristic vibrational absorptions in this waveband. Specifically, the C-O stretching vibration of the ester group and the bending vibration of the long-chain alkyl group cause changes in the absorption peaks of WPA in the 0~1353.91 cm^−1^ waveband compared with VA. These changes reflect the spectral response of the interaction between modifier molecules and asphalt molecules in this waveband.

A comparison of the characteristic peak intensities between VA and WPA in the wavenumber ranges of 2795.34~3006.06 cm^−1^ and 1353.91~1542.03 cm^−1^ reveals that the absorption peak intensity of WPA in the characteristic wavenumber ranges is significantly higher than that of VA. As one of the heavy components in the asphalt colloid system, asphaltene content exerts a decisive influence on key performance parameters of asphalt, such as viscoelasticity and temperature sensitivity. The increase in the characteristic peak intensity of WPA intuitively reflects a substantial rise in the relative proportion of asphaltene components within it. From the perspective of modification mechanism, this phenomenon can be attributed to the preparation process of WPA, the large amount of EVA and PE added to WPA interact physically with asphalt. Through intermolecular adsorption and dispersion effects, EVA and PE absorb the light components (e.g., saturates and aromatics) in asphalt. As the proportion of light components decreases, the relative content of heavy components such as asphaltene increases accordingly, ultimately manifesting as a change in the intensity of characteristic peaks in the infrared spectrum.

### 3.4. TLC-FID Test

**Figure 7:**
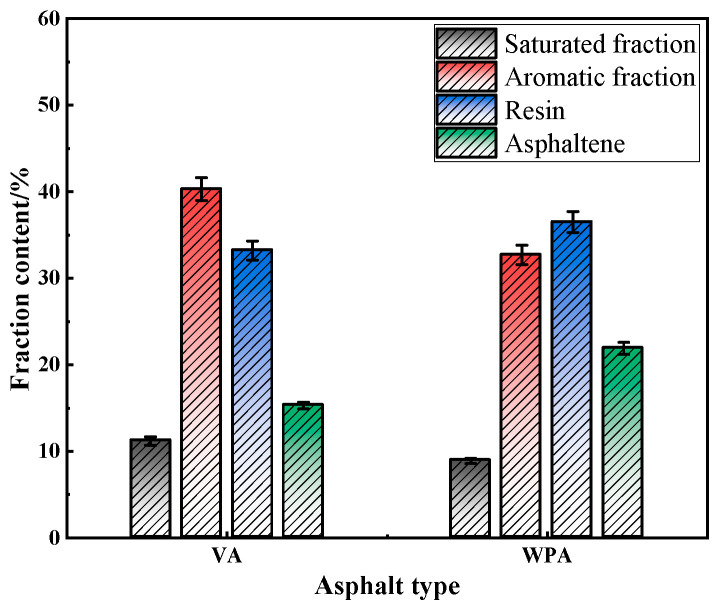
The component contents of several asphalts.

To further evaluate the effect of modification on the internal components of asphalt, and thereby verify the analytical results of the FTIR test, this study analyzed the internal components of several types of asphalt using the TLC-FID test. The results are presented in Figure 7.

As shown in Figure 7, there are significant differences in the contents of the four major components—saturates, aromatics, resins, and asphaltenes—between VA and WPA. Specifically, the saturate content of VA is higher than that of WPA. As a component in asphalt with small molecular weight and simple structure, saturates affect the fluidity and low-temperature performance of asphalt. The lower saturate content may result in WPA having weaker fluidity than VA at low temperatures, but it also provides room for the performance of other components to be exerted. Meanwhile, the aromatic content of VA is significantly higher than that of WPA. Aromatics play a role in dissolving asphaltenes and resins; the reduction in aromatic content of WPA reflects that during the modification process, some aromatics may have participated in interactions with the modifier, or the relative proportion of aromatics has changed due to the introduction of the modifier. In terms of resins and asphaltenes, the resin content of WPA is significantly higher than that of VA. Resins play a role in stabilizing the colloidal structure of asphalt, they can adsorb around asphaltenes and prevent asphaltenes from flocculating and precipitating. The increase in resin content helps enhance the stability of the internal structure of asphalt and improve properties such as its adhesion and flexibility. Additionally, the asphaltene content of WPA is also higher than that of VA. As the component in asphalt with the largest molecular weight and most complex structure, asphaltenes are crucial for the high-temperature stability of asphalt. The increase in asphaltene content can enhance WPA’s deformation resistance in high-temperature environments and reduce the occurrence of distresses such as high-temperature rutting. In summary, compared with VA, WPA exhibits a significant change in component distribution: the contents of saturates and aromatics decrease, while the contents of resins and asphaltenes increase. This change in components is closely related to the effect of EVA and PE. When the modifier is blended with asphalt, on one hand, it may affect the existing form and proportion of the original components of asphalt through physical or chemical interactions. This causes more light components to participate in interactions with the modifier, relatively increasing the proportion of heavy components. On the other hand, the increase in the contents of resins and asphaltenes helps optimize the colloidal structure of asphalt: it not only enhances high-temperature stability but also ensures the balance of the overall performance of asphalt through the stabilizing effect of resins. This is consistent with the conclusion of improved modified asphalt performance verified by previous tests such as FTIR, further indicating that the modification effectively changes the internal components of asphalt, thereby improving the performance of asphalt.

## 4. Conclusions

In this study, a new type of modified asphalt was prepared using composite modification technology. The service performance and modification mechanism of asphalt before and after modification were characterized through conventional performance tests, rheological tests, and microscopic tests. The main conclusions are as follows:

(1) Orthogonal tests were conducted to analyze the formula of composite modified asphalt, and the recommended dosage was determined as 5% WCO, 5% EVA, and 5% PE.
(2) Compared with VA, the high-temperature complex modulus of WPA is increased, the phase angle is decreased, and its high-temperature performance grade reaches PG 70. In addition, WPA shows improvements in rutting resistance, fatigue performance, and low-temperature performance. The modification effectively optimizes the rheological properties of asphalt.
(3) The incorporation of several modified materials causes changes in the internal components of asphalt, the contents of light components in asphalt decrease, while the proportion of heavy components increases. This component change further improves asphalt’s performance in terms of high-temperature stability, fatigue resistance, and low-temperature crack resistance.

This study clarifies the mechanism for improving the rheological properties of composite modified asphalt made from recycled plastics and waste edible oil, but there are limitations: the tests were conducted only under standard indoor conditions, without considering the complex working conditions in actual engineering. The impact of composition fluctuations from different sources of recycled materials on the modification effect was also not explored. Additionally, there is a lack of analysis on long-term aging performance and the economic and environmental impacts throughout the entire lifecycle. In the future, environmental-load-coupled aging tests under real-world conditions can be conducted, the pretreatment processes for recycled materials can be optimized, long-term microstructural degradation mechanisms can be revealed, and economic and environmental assessments can be incorporated to provide systematic support for the technology’s promotion.

## Figures and Tables

**Table 1:** Basic performance of VA.

Test	Index	Value	Requirement	Test Method
Penetration	0.1 mm	83.1	80~100	JTG E20-2011 T 0604 [20]
Ductility	cm	>100	>100	JTG E20-2011 T 0605
Softening point	°C	44.5	>44	JTG E20-2011 T 0606
RTFOT (163 °C, 85 min)	Mass loss rate/%	−0.35	≤±0.8	JTG E20-2011 T 0603
	Penetration ratio/%	73.1	≥57	JTG E20-2011 T 0610
	Residual ductility/cm	56.5	≥20	JTG E20-2011 T 0604

**Table 2:** The properties of several modified materials.

Material Type	Properties	Test Value	Test Method
WCO	Density, g/cm^−3^	0.94	GB/T 5526-2024 [21]
	Viscosity, Pa·s	0.05	GB/T 22235-2008 [22]
	Flash point, °C	243	GB/T 21789-2025 [23]
	Moisture content, %	0.15	GB 5009.236-2016 [24]
	Exterior	Brown oily liquid	
PE	Density, g/cm^−3^	1.05	GB/T 1033.1-2008 [25]
	Melting point, °C	105	YY/T 0815-2010 [26]
	Flash point, °C	270	GB/T 9343-2008 [27]
	Exterior	Colorless transparent particle	
EVA	Density, g/cm^−3^	0.945	GB/T 1033.1-2008
	Melting point, °C	103	GB/T 15182-2020 [28]
	Thermal decomposition temperature, °C	241	GB/T 33047.1-2016 [29]
	Exterior	Transparent particle	

**Table 3:** Factor level table.

Level	(A) WCO/%	(B) EVA/%	(C) PE/%
1	5	3	4
2	7	4	5
3	9	5	6

**Table 4:** The results of orthogonal test.

Number	Factor			Evaluation Indexes			
	A	B	C	Penetration/0.1 mm	Softening Point/°C	Ductility/cm	Viscosity/Pa·s
1	1	1	1	83.5	45.1	7	0.367
2	1	2	3	79.6	48.3	9.1	0.765
3	1	3	2	77.4	50.1	11.2	0.931
4	2	1	3	82.6	45.7	7.3	0.389
5	2	2	2	83.8	44.9	6.8	0.351
6	2	3	1	81.6	46.2	7.9	0.422
7	3	1	2	85.7	43.5	6.1	0.339
8	3	2	1	84.1	44.7	6.6	0.347
9	3	3	3	83.6	45.1	6.9	0.369
K1	80.2	83.9	83.1	Intuitive analysis of penetration			
K2	82.7	82.5	82.3				
K3	84.5	80.9	81.9				
R	4.3	3.1	1.1				
Solution	A_3_	B_1_	C_1_				
K1	47.8	44.8	45.3	Intuitive analysis of softening point			
K2	45.6	46.0	46.2				
K3	44.4	47.1	46.4				
R	3.4	2.4	1.0				
Solution	A_1_	B_3_	C_3_				
K1	9.1	6.8	7.2	Intuitive analysis of ductility			
K2	7.3	7.5	8.0				
K3	6.5	8.7	7.8				
R	2.6	1.9	0.9				
Solution	A_1_	B_3_	C_2_				
K1	0.7	0.4	0.4	Intuitive analysis of viscosity			
K2	0.4	0.5	0.5				
K3	0.4	0.6	0.5				
R	0.3	0.2	0.2				
Solution	A_1_	B_3_	C_2_				

**Table 5:** The test results of several asphalts.

Properties	Index	A_1_B_3_C_1_	A_1_B_3_C_2_	A_1_B_3_C_3_
Penetration	(25 °C)/0.1 mm	79.1	77.4	76.2
Ductility	(5 °C)/cm	48.3	50.1	51.1
Softening point	°C	9.9	11.2	11.6
Viscosity	Pa·s	0.797	0.931	1.012

## Data Availability

The original contributions presented in this study are included in the article. Further inquiries can be directed to the corresponding authors.

## References

[1] Zhou J., Li J., Liu G., Yang T., Zhao Y. (2021). Recycling aged asphalt using hard asphalt binder for hot-mixing recycled asphalt mixture. Appl. Sci..

[2] Fry J., Schandl H. (2024). Simulating waste plastic flows in Australia. Resources. Conserv. Recycl..

[3] Ishimura Y., Nomura K., Ichinose D. (2025). Does simplification of plastic waste separation promote plastic recycling?. J. Mater. Cycles Waste Manag..

[4] Briassoulis D., Babou E., Hiskakis M., Scarascia G., Picuno P., Guarde D., Dejean C. (2013). Review, mapping and analysis of the agricultural plastic waste generation and consolidation in Europe. Waste Manag. Res..

[5] Kumi-Larbi Jnr A., Galpin R., Manjula S., Lenkiewicz Z., Cheeseman C. (2022). Reuse of waste plastics in developing countries: Properties of waste plastic-sand composites. Waste Biomass Valorization.

[6] Sharma D.K., Bapat S., Brandes W.F., Rice E., Castaldi M.J. (2019). Technical feasibility of zero waste for paper and plastic wastes. Waste Biomass Valorization.

[7] Yang X., Jiang X., He Z., Zhang X. (2010). Modified Asphalt From Domestic Waste Old Plastic. Intell. Autom. Soft Comput..

[8] Dalhat M.A., Al-Abdul Wahhab H. (2017). Performance of recycled plastic waste modified asphalt binder in Saudi Arabia. Int. J. Pavement Eng..

[9] Boom Y.J., Enfrin M., Swaney M., Masood H., Pramanik B.K., Robert D., Giustozzi F. (2023). Engineering properties, microplastics and emissions assessment of recycled plastic modified asphalt mixtures. Sci. Total Environ..

[10] Lu D.X., Enfrin M., Boom Y.J., Giustozzi F. (2023). Future recyclability of hot mix asphalt containing recycled plastics. Constr. Build. Mater..

[11] Dalhat M., Al-Abdul Wahhab H.I., Al-Adham K. (2019). Recycled plastic waste asphalt concrete via mineral aggregate substitution and binder modification. J. Mater. Civ. Eng..

[12] Hao G., He M., Lim S.M., Ong G.P., Zulkati A., Kapilan S. (2024). Recycling of plastic waste in porous asphalt pavement: Engineering, environmental, and economic implications. J. Clean. Prod..

[13] de Melo J.V.S., Tonial M., Manfro A.L. (2025). Impact of incorporating latex glove fragments on the rheological and mechanical properties of a conventional asphalt matrix. Constr. Build. Mater..

[14] Ma Y., Demchuk Z., Polaczyk P., Zhou H., He Q., Baumgardner G.L., Huang B. (2025). Reactive extrusion of waste plastics with compatibilizer and lightly pyrolyzed crumb rubber for asphalt modification. Transp. Res. Rec..

[15] Mamun A.A., Al-Abdul Wahhab H., Dalhat M. (2020). Comparative evaluation of waste cooking oil and waste engine oil rejuvenated asphalt concrete mixtures. Arab. J. Sci. Eng..

[16] Li H., Zhang F., Feng Z., Li W., Zou X. (2021). Study on waste engine oil and waste cooking oil on performance improvement of aged asphalt and application in reclaimed asphalt mixture. Constr. Build. Mater..

[17] Zhong Q., Zhang H., Sun J., Zhu J., Fan X. (2025). Optimizing asphalt recycling through characterization of waste cooking oils. Constr. Build. Mater..

[18] Bardella N., Facchin M., Fabris E., Baldan M., Beghetto V. (2024). Waste cooking oil as eco-friendly rejuvenator for reclaimed asphalt pavement. Materials.

[19] Mamun A.A., Al-Abdul Wahhab H. (2020). Comparative laboratory evaluation of waste cooking oil rejuvenated asphalt concrete mixtures for high contents of reclaimed asphalt pavement. Int. J. Pavement Eng..

[20] (2011). Standard Test Methods of Bitumen and Bituminous Mixtures for Highway Engineering.

[21] (2024). Animal and Vegetable Fats and Oils—Determination of Relative Density.

[22] (2008). Determination for Viscosity of Liquids.

[23] (2025). Determination of Flash Point—Abel Closed-Cup Method.

[24] (2016). National Food Safety Standard—Determination of Moisture and Volatile Substances in Animal and Vegetable Oils and Fats.

[25] (2008). Plastics. Methods for Determining Density of Non-Celluar Plastics. Part 1: Immersion Method, Liquid Pyknometer Method and Titration Method.

[26] (2010). Standard Test Method for Measurement of Enthalpy of Fusion, Percent Crystallinity, and Melting Point of Ultra-High-Molecular Weight Polyethylene by Means of Differential Scanning Calorimetry.

[27] (2008). Test Method for Flammability of Plastics.

[28] (2020). Linear Low Density Polyethylene Resin.

[29] (2016). Plastics-Thermogravimetry(TG) of Polymers Part 1: General Principles.

[30] Cong P., Hao H., Luo W. (2019). Investigation of carbonyl of asphalt binders containing antiaging agents and waste cooking oil using FTIR spectroscopy. J. Test. Eval..

[31] Yuechao Z., Meizhu C., Shaopeng W., Qi J. (2022). Rheological properties and microscopic characteristics of rejuvenated asphalt using different components from waste cooking oil. J. Clean. Prod..

[32] Zhong L., Zhang Y., Wang T., Ji Y., Norris P., Pan W.-P. (2019). Optimized methods for preparing activated carbon from rock asphalt using orthogonal experimental design. J. Therm. Anal. Calorim..

[33] Wei H., Wang Y., Li J., Zhang Y., Qian G. (2023). Optimization design of cold patching asphalt liquid based on performance experiments and statistical methods. Constr. Build. Mater..

[34] (2019). Standard Method of Test for Multiple Stress Creep Recovery (MSCR) Test of Asphalt Binder Using a Dynamic Shear Rheometer (DSR).

[35] (2012). Standard Method of Test for Estimating Fatigue Resistance of Asphalt Binders Using the Linear Amplitude Sweep.

[36] (2019). Standard Method of Test for Determining the Flexural Creep Stiffness of Asphalt Binder Using the Bending Beam Rheometer (BBR).

[37] (2019). General Rules for Infrared Analysis.

[38] (2025). Standard Test Methods of Asphalt And Asphalt Mixture for Highway Engineering.

[39] Al-Khateeb G.G., Ramadan K.Z. (2015). Investigation of the effect of rubber on rheological properties of asphalt binders using superpave DSR. KSCE J. Civ. Eng..

[40] Zhang W., Ma T., Xu G., Huang X., Ling M., Chen X., Xue J. (2018). Fatigue resistance evaluation of modified asphalt using a multiple stress creep and recovery (MSCR) test. Appl. Sci..

[41] Yan C., Yuan L., Yu X., Ji S., Zhou Z. (2022). Characterizing the fatigue resistance of multiple modified asphalts using time sweep test, LAS test and elastic recovery test. Constr. Build. Mater..

[42] Yu H., Wu S., Chen A., Li Y. (2023). Modification mechanism and technical performance of recycled PE-modified asphalt. Sustainability.

[43] Zhao Z., Wu S., Xie J., Yang C., Yang X., Chen S., Liu Q. (2023). Recycle of waste tire rubber powder in a novel asphalt rubber pellets for asphalt performance enhancement. Constr. Build. Mater..

[44] Alghrafy Y.M., Abd Alla E.S.M., El-Badawy S.M. (2021). Rheological properties and aging performance of sulfur extended asphalt modified with recycled polyethylene waste. Constr. Build. Mater..

[45] Meng F., Ma S., Muhammad Y., Li J., Sahibzada M., Chi F. (2020). Analysis of virgin asphalt brands via the integrated application of FTIR and gel permeation chromatography. Arab. J. Sci. Eng..

[46] Zeiada W., Al-Khateeb G., Hajj E.Y., Ezzat H. (2024). Rheological properties of plastic-modified asphalt binders using diverse plastic wastes for enhanced pavement performance in the UAE. Constr. Build. Mater..

[47] Shen B.X., Zhao L.M., Liu F.X. (1994). TLC/FID group compositional analytical method of micro-amounts of asphalts and its application. Fuel Sci. Technol. Int..

[48] Bastola N.R., Teixeira J.E.S.L. (2025). Low-temperature cracking assessment of high-RAP mixtures modified with waste plastics and vegetable oil using multi-test performance-based parameters. Constr. Build. Mater..

